# Impact of Hepatitis B Infection on Patient and Graft Survival After Kidney Transplantation

**DOI:** 10.3390/jcm14062124

**Published:** 2025-03-20

**Authors:** Anissa Paschereit, Vivien Greese, Kayo Sakurayama, Michael Duerr, Fabian Halleck, Lutz Liefeldt, Mira Choi, Klemens Budde, Marcel G. Naik

**Affiliations:** 1 Department of Nephrology and Medical Intensive Care, Charité-Berlin University Medicine, Charitéplatz 1, 10117 Berlin, Germany; vivien.greese@charite.de (V.G.); kayo.sakurayama@charite.de (K.S.); michael.duerr@boehringer-ingelheim.com (M.D.); fabian.halleck@charite.de (F.H.); lutz.liefeldt@charite.de (L.L.); mira.choi@charite.de (M.C.); klemens.budde@charite.de (K.B.); marcel.naik@charite.de (M.G.N.); 2 Boehringer Ingelheim, Friedrichstraße 70, 10117 Berlin, Germany

**Keywords:** hepatitis B infection, kidney transplantation, patient survival, graft survival, survival analysis, HbsAg, Anti-Hbc

## Abstract

**Objectives**: Chronic Hepatitis B virus (HBV) infection is a significant global health issue, with dialysis patients at increased risk and reduced response to HBV vaccination. The effects of HBV serological status on kidney transplant outcomes, particularly for patients with resolved or inactive HBV infection, needs more data, especially from current era. This study evaluated the impact of chronic and non-active HBV infection on patient and graft survival after kidney transplantation. **Methods**: Retrospective analysis was conducted of kidney-only transplant recipients at our center from 1 January 1990 to 31 August 2019 (end of observation). Patients were grouped by their HBV serostatus before transplantation into three categories: HBV negative (HBsAg−/Anti-Hbc−), non-active HBV infection (HbsAg−/Anti-Hbc+) and chronic HBV infection (HbsAg+/Anti-Hbc+). Primary outcomes included patient survival, graft survival, and overall graft and patient survival, analyzed using Kaplan–Meier (KM) curves, log-rank tests, Restricted mean survival times (RMST), and Accelerated failure time (AFT) models. **Results**: Among 2490 patients, 2197 were HBV negative, 218 had non-active HBV, and 75 had chronic HBV. Over a mean follow-up of 8.1 years, mortality and graft failure rates were highest in chronic HBV patients (49% and 37%), followed by non-active HBV (39% and 29%) and HBV-negative patients (30% and 20%). KM analysis revealed significantly lower overall survival rates for chronic HBV and non-active HBV groups compared to HBV-negative patients (*p* = 0.006). RMST confirmed significant reductions in survival for the non-active group (12.57 vs. 14.17 years, *p* = 0.007). Cox regression and AFT models identified older recipient/donor age, Hepatitis-C-virus coinfection, and broad antigen mismatches as negative predictors, while living donors improved outcomes. **Conclusions**: While unadjusted Kaplan–Meier curves and RMST analysis suggested differences in patient and graft survival, further thorough multivariable AFT analysis did not show a significant association between non-active or chronic HBV infection and patient or graft survival after kidney transplantation.

## 1. Introduction

Chronic Hepatitis B (HBV) infection affects more than 254 million people worldwide, making it a major global health burden despite the availability of antiviral drugs and vaccines [1,2,3]. In Germany, the prevalence of hepatitis B is relatively low, with approximately 5.1% of adults showing serological evidence of current or past infection [4,5]. Hepatitis B virus infection typically presents in two serological phases: an early infection phase characterized by positive Hepatitis B surface antigen (HBsAg+) and negative hepatitis B core antibody (HbcAb−), which may become chronic; and a resolving non-active infection phase, where HBsAg becomes negative, and hepatitis B core antibody (Anti-HBc) becomes positive (HBsAg−/Anti-HBc+) [2,6,7].

Germany implemented routine HBV testing of blood products in 1978, significantly improving blood safety through HBsAg detection. Further improvements in the 1990s, including the introduction of nucleic acid amplification testing, reduced the risk of HBV transmission [8]. Since the enactment of the Transfusion Act in 1998, Hepatitis B screening of blood products was mandatory [9]. As a result, patients who underwent transplantation in the 1990s presented with a higher rate of hepatitis B due to historical screening limitations [10].

In the present era, HBV infection has become controllable using antiviral drugs and protective vaccination. However, it remains a major threat in patients with end-stage renal disease undergoing dialysis, affecting from 1.3% up to 14.6% across different regions [11]. These patients face increased risks of HBV infection due to potential exposure to the virus during hemodialysis and impaired immune responses due to chronic kidney disease [12,13,14]. These immune deficiencies have been partially attributed to the accumulation of uremic retention solutes [15,16]. As a result, higher vaccination doses are recommended. Despite these measures, chronic hemodialysis patients frequently exhibit low antibody levels, leading to inadequate protection against HBV infection [17,18,19].

Previous studies indicate that patients with chronic HBV infection have decreased survival rates after kidney transplantation [20,21,22,23,24]. HBsAg-positive transplant recipients tend to have poorer patient and graft survival outcomes compared to those without HBV infection. Nonetheless, the specific impact of HBV infection on post-transplantation outcomes remains a subject of debate. Transplant-related immunosuppression in recipients with HBV infection may increase viral replication, which in turn can accelerate progression of liver disease like cirrhosis and hepatocellular carcinoma as well as development of HBV-related renal diseases such as membranous nephropathy and membranoproliferative glomerulonephritis [25]. These conditions could negatively influence both graft and patient survival.

Given the significance of this prevalent infectious disease among kidney transplant recipients, its effect on post-transplantation outcomes holds substantial importance. While the impact of chronic hepatitis B on these outcomes has been studied, the effects of a previously resolved hepatitis B infection are less clear and not fully understood yet. There is a potential of post-transplantation reactivation of HBV under immunosuppression in patients with positive Anti-HBc status at the time of transplantation [26]. However, data on the long-term outcomes of these patients are sparse [27]. Further investigation into both patient- and transplant-related factors is necessary to determine their potential contribution to post-transplant outcomes.

The aim of this study was to evaluate how graft and patient survival after kidney transplantation are influenced by the serological HBV status. In addition to examining the impact of chronic hepatitis B, the study aimed to explore whether inactive hepatitis B at time of transplantation impacts patient and graft outcomes. We hypothesized that both chronic and inactive hepatitis B statuses significantly affect patient and graft survival following kidney transplantation.

## 2. Materials and Methods

### 2.1. Subjects

We retrospectively reviewed data from kidney-only transplant recipients in our centre between 1 January 1990 and 31 August 2019 (end of observation). Grafts were obtained from both living and deceased donors. The retrospective analysis was performed on prospectively collected data from our nephrological transplant database (TBase) at Charité Campus Mitte and Charité Campus Virchow [28]. Only patients with complete information on Hepatitis B serological status (HBsAg and anti-HBc) prior to transplantation were included in the analysis. Children and patients that received multiorgan transplantations were excluded. All patients gave informed consent on retrospective scientific analysis of their data. Our study was conducted in accordance with the ethical approval granted by the institutional ethics committee (EA1/132/20), issued on 17 November 2020.

### 2.2. Clinical and Laboratory Data Collection

Data retrieved from TBase encompassed recipient related data such as gender, blood group, anti-HBs level prior to transplantation, underlying disease, and death reason. Additionally, donor related data such as gender, blood group, donor BMI, donor type (living vs. deceased) were included. Dialysis specific data such as the type of dialysis (peritoneal dialysis vs. haemodialysis) and the date when dialysis was initiated, was also part of the retrieved data. Moreover, immunological data consisted of values such as Hepatitis B serostatus, Panel reactive antibodies (PRA), number of broad antigen mismatches, Hepatitis C virus (HCV) antibodies, cytomegalovirus (CMV) antibodies. The transplantation related data comprising various details, including the transplantation date, the development of primary graft function (defined as urinary output within the surgery), cold ischemia time, residual diuresis and recipient side (right or left iliac fossa).

### 2.3. Hepatitis B Status Definition

Patients were grouped by their serostatus prior to kidney transplantation into Hepatitis B negative, non-active Hepatitis B infection and chronic Hepatitis B infection. Patients with negative HBsAg and negative anti-HBc were grouped as Hepatitis B negative (HBsAg−/HbcAb−). Non-active HBV infection was defined by negative HBsAg and positive anti-HBc-antibodies (HBsAg−/HbcAb+) and chronic Hepatitis B infection by positive HBsAg and positive anti-HBc (HBsAg+/HbcAb+).

### 2.4. Outcome Measures

The outcome measures of interest included the following: (1) patient survival, taken from the time of transplantation until death; (2) graft survival, taken from the time of transplantation until graft failure; and (3) overall survival, defined as the composite endpoint of patient death and graft failure, taken from the time of transplantation until death or graft failure. Graft failure was defined as the need for the recipient to return to dialysis or undergo graft removal and re-transplantation. For all outcomes, patients were censored at their last observation if the outcome was not reached. If the last observation date was beyond the study period, patients were censored on 31 August 2019. Incomplete follow-up was defined as a time frame between the end of the observation period and the censoring date that exceeded 1.5 years.

### 2.5. Statistical Analysis

Continuous variables were reported as the mean value along with the standard deviation. Categorial variables were summarized as frequencies and percentages. Kaplan–Meier survival curves were plotted to visualize the impact of Hepatitis-B-status on the time of event. Log-rank test was employed to statistically compare the survival curves. Restricted Mean Survival Times were calculated to estimate the life expectancy for patients based on their Hepatitis B group and to compare them by looking at the difference in restricted mean survival times. Cox proportional hazard regression model was applied to investigate potential interactions among the predictors (patient and transplant characteristics) in relation to the outcome. Recipient and transplant-related variables such as recipient age, donor age, cold ischemia time, Hepatitis C virus antibodies, donor type, Cytomegalovirus antibodies and broad antigen mismatches were considered as confounding variables in the analysis. The variables used in the analysis had a completeness of 99.4%. As the Kaplan–Meier-Analysis indicated that the proportional hazards assumption was not met, accelerated failure time models were additionally calculated to account for this deviation. The AFT model was used to analyse the relationship between survival times and predictor variables for the three outcomes. The predictor variables taken into consideration were the same as in the Cox proportional hazard model. Statistical analysis was conducted using R Studio (version RStudio 2022.07.0+548). Differences with a *p*-value < 0.05 were considered statistically significant.

## 3. Results

We identified 3.663 adult (>18 years) kidney-only transplant recipients between 1 January 1990 and 31 August 2019 in our centre. In 2490 patients, Hepatitis B status at the time of transplantation was retrievable, of which 2197 patients were categorized into the “Hepatitis B negative” group, 218 patients into the “non-active Hepatitis B” group and 75 patients into the “chronic Hepatitis B” group.

### 3.1. Demographic Characteristics

The clinical and transplant-specific characteristics of the recipients and donors are reflected in Table 1. The mean age was around 50 years (range 18–86 years), while patients with chronic Hepatitis B infection were the youngest with a mean age of 46 years, and patients with non-active HBV infection were the oldest (mean age: 53 years). The prevalence of HCV coinfection was approximately seven times higher in individuals with either non-active or chronic HBV infection compared to those who were Hepatitis B negative. Patients with negative HBV serostatus exhibited a slightly lower participation rate in haemodialysis and a higher incidence of peritoneal dialysis or no dialysis prior to transplantation compared to the remaining patient groups. The mean time on dialysis was remarkably lower in patients with negative HBV serostatus. Considering the transplantation eras, the majority of patients in all three groups received their transplants between 2000 and 2009. Among patients with negative or non-active HBV status, the second highest number of transplants occurred in the most recent era from 2010 to 2019. In contrast, for patients with chronic HBV infection, transplants were more frequently performed at the beginning of our study period between 1990 and 1999. The distribution of broad antigen mismatches exhibited similarities across the different patient groups. Recipients with chronic HBV infection had a higher level of panel reactive antibodies compared to the other groups of patients. Although most grafts were sourced from deceased donors overall (76%), a comparative analysis across the three patient groups revealed that recipients with negative Hepatitis B status received a slightly higher proportion of living donor transplants (25%) in contrast to those with chronic HBV infection (17%) or non-active HBV infection (19%). Among the patient groups, those with chronic HBV infection experienced the longest cold ischemia time (13.1 h), while recipients with negative serostatus had the shortest duration (9.1 h). The mean age of the donors across all groups was 52 years. Among patients with chronic HBV infection, the donors were the youngest, with a mean age of 46 years, compared to a mean donor age of 51 years in patients with non-active HBV infection and 53 years in HBV negative patients.

### 3.2. Clinical Outcomes

Patient outcomes are presented in Table 2. Overall, 31% of the patients died, 21% had a graft failure, and 42% died and/or had graft failure. In a mean follow-up duration of 8.1 years, kidney transplant recipients with negative Hepatitis B status showed a reduced number of deaths (30%) compared to the recipients with a non-active Hepatitis B (39%) or with a chronic Hepatitis B (49%). The rates of graft failure among kidney transplant recipients were 20% for those with negative Hepatitis B status, 29% for those with non-active Hepatitis B, and 37% for those with chronic Hepatitis B. The occurrence of death and/or transplant failures was reduced among kidney transplant recipients with negative Hepatitis B status (40%) compared to those with non-active (53%) or chronic (68%) Hepatitis B status. Of the initially identified 2490 patients with retrievable Hepatitis B status, a total of 194 (7.8%) were lost to follow-up, as their last documented visit occurred 1.5 years before censoring, which was used as the threshold to define loss to follow-up.

In conclusion, kidney transplant recipients with non-active or chronic Hepatitis B infection showed higher rates of death, graft failure, and the combined event of death and/or graft failure compared to those with negative Hepatitis B status.

### 3.3. Kaplan–Meier-Analysis

**Figure 1:**
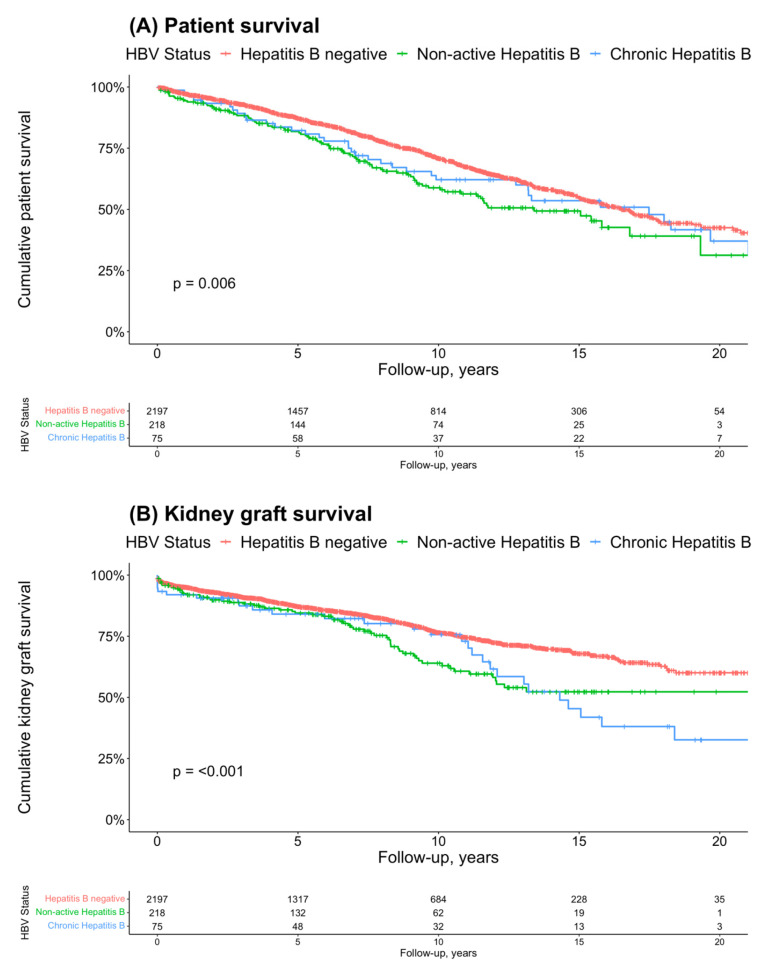

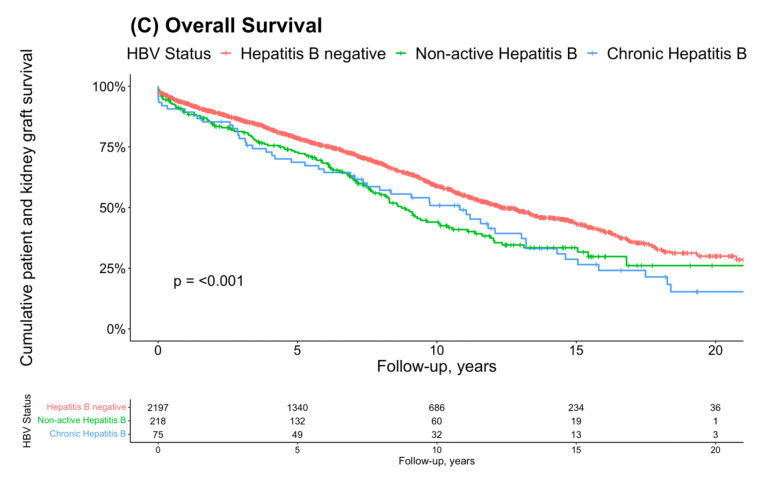
(**A**) Kaplan–Meier analysis for the endpoint patient survival and tabular representation of the patients under observation at 0, 5, 10, 15, 20 years, *p* = 0.006; (**B**) Kaplan–Meier analysis for the endpoint graft survival and tabular representation of the grafts under observation at 0, 5, 10, 15, 20 years, *p* < 0.001; (**C**) Kaplan–Meier analysis for the combined endpoint of patient and graft survival and tabular representation of the patients under observation at 0, 5, 10, 15, 20 years, *p* < 0.001.

Patient survival times were compared among the Hepatitis B groups using Kaplan–Meier curves (Figure 1A) Log-rank test results revealed a statistically significant difference in survival between the three groups (*p* = 0.006). The cumulative unadjusted 10-year survival rate of kidney transplant recipients with chronic HBV was 62.1%; for those with non-active HBV, it was 58.8%; and for recipients with negative HBV serology, it was 70.9% (Table A1a).

The Kaplan–Meier curves for comparison of graft survival in the three Hepatitis B groups are shown in Figure 1B. The results of the log-rank test indicated a statistically significant divergence in survival rates among the three groups (*p* < 0.001). Over 10 years, 75.7% of the patients and grafts survived in the chronic HBV group, 64.0% in the non-active HBV group, and 76.5% in the “negative” group in an unadjusted analysis (Table A1b).

The Kaplan–Meier curves for comparison of overall survival in the three Hepatitis B groups are shown in Figure 1C. The unadjusted log-rank test outcomes demonstrated a statistically significant variation in survival rates across the three groups (*p* < 0.001). The cumulative unadjusted 10-year overall patient graft survival rates of the kidney transplant recipients with chronic HBV, non-active HBV and negative serology were 50.9%, 44.0%, and 59.0%, respectively (Table A1c).

In summary, unadjusted Kaplan–Meier analysis revealed statistically significant differences in survival, graft survival, and overall survival among kidney transplant recipients, with those having negative Hepatitis B serology consistently showing better 5-, 10-, and 20-year outcomes compared to those with non-active or chronic Hepatitis B. However, the crossing of the curves indicates a potential violation of the proportional hazards assumption.

### 3.4. Restricted Mean Survival Times

**Figure 2:**
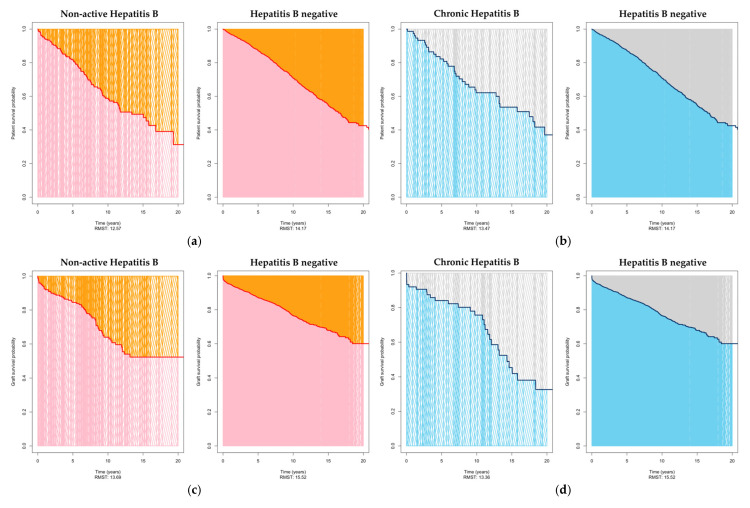

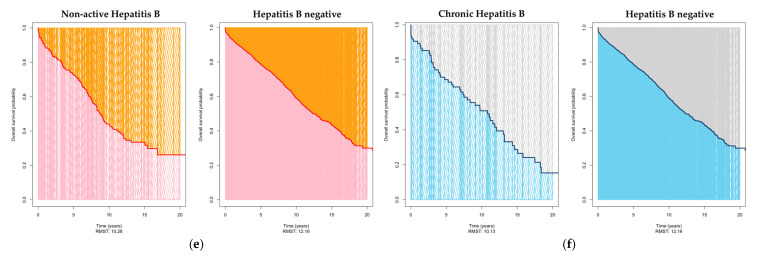
Comparison of Restricted Mean Survival Times (RMST): (**a**) for patient survival: non-active Hepatitis B vs. Hepatitis B negative, *p* = 0.007; (**b**) for patient survival: chronic Hepatitis B vs. Hepatitis B negative, *p* = 0.427; (**c**) for graft survival: non-active Hepatitis B vs. Hepatitis B negative, *p* = 0.005; (**d**) for graft survival: chronic Hepatitis B vs. Hepatitis B negative, *p* = 0.018; (**e**) for overall survival: non-active Hepatitis B vs. Hepatitis B negative, *p* = 0.001; (**f**) for overall survival: chronic Hepatitis B vs. Hepatitis B negative, *p* = 0.016.

Restricted Mean Survival Times (RMST) represent the area under the survival curve up to a specific time point. We quantified the average survival within 20 years. The RMST differences are graphically presented in Figure 2a,b for patient survival, in Figure 2c,d for graft survival, and in Figure 2e,f for overall survival.

Figure 2a illustrates the contrast in unadjusted RMST between those patients with non-active Hepatitis B infection and those who tested negative for Hepatitis B. For patients with non-active Hepatitis B infection, the calculated RMST was 12.57 years (95% CI: 11.45–13.69 years). In contrast, patients who were Hepatitis B negative prior to transplantation exhibited a significantly longer RMST of 14.17 years (95% CI: 13.83–14.51 years; *p* = 0.007). Figure 2b provides a visual representation of the comparison in Restricted Mean Survival Times between those patients with chronic Hepatitis B infection and those with negative Hepatitis B test results. Among patients with chronic Hepatitis B infection, the computed RMST stood at 13.47 years (95% CI: 11.80–15.15 years). Conversely, patients who tested negative for Hepatitis B prior to transplantation exhibited an RMST of 14.17 years (95% CI: 13.83–14.51 years), resulting in a non-significant difference (*p* = 0.427).

As shown in Figure 2c, the analysis of the unadjusted RMST for transplant survival revealed that patients with non-active Hepatitis B infection had an estimated RMST of 13.69 years (95% CI: 12.49–14.90 years), while patients with a negative serostatus exhibited an RMST of 15.52 years (95% CI: 15.15–15.88 years). This suggests that, on average, transplanted organs in the non-active group lasted 1.82 years longer than those in the negative group within the specified time frame, and the difference in graft survival time is proven to be significant (*p* = 0.005). In Figure 2d our unadjusted examination of RMST for transplant survival uncovered notable distinctions. Patients with chronic Hepatitis B infection demonstrated an estimated RMST of 13.36 years (with a 95% CI ranging from 11.61 to 15.12 years), while individuals testing negative for Hepatitis B had an RMST of 15.52 years (95% CI: 15.15–15.88 years; *p* = 0.018).

The estimated RMST for the overall survival was 10.28 years (95% CI: 9.19–11.37 years) for patients with non-active Hepatitis B infection (Figure 2e), compared to patients with a negative serostatus showing an RMST of 12.16 years (95% CI: 11.81–12.52 years). The results indicated a statistically significant difference between the negative and non-active group (*p* = 0.001), suggesting that the overall life expectancy of these groups has a difference of 1.88 years. As shown in Figure 2f patients with chronic Hepatitis B infection demonstrated a RMST of 10.13 years (95% CI: 8.52–11.75 years), showing significant difference compared to the HBV-negative cohort (*p* = 0.016).

The RMST analysis demonstrated that kidney transplant recipients with non-active Hepatitis B infection had significantly shorter survival times for patient survival, graft survival, and overall survival compared to those with negative Hepatitis B status. In contrast, the differences in patient survival for recipients with chronic Hepatitis B infection were less pronounced and not significant.

### 3.5. Cox Proportional Hazards Model

We performed a Cox regression analysis to examine the impact of various factors on patient survival, graft survival and overall survival. The analysis identified several factors that significantly influence the three mentioned endpoints. However, as the proportional hazards assumption was not met, we additionally employed an Accelerated Failure Time (AFT) model for a more accurate and reliable analysis of these data.

The Cox regression for patient survival (Table A2a) identified that older recipient age (Exp(B) = 1.066, *p* < 0.001), positive HCV antibody status (Exp(B) = 1.542, *p* = 0.004), receiving an organ from a deceased donor (Exp(B) = 0.554, *p* < 0.001) and increased number of broad antigen mismatches (Exp(B) = 1.079, *p* = 0.003) significantly affected graft survival.

The Cox regression model for graft survival (Table A2b) also showed that several variables have a significant impact. These include: older age of the donor (Exp(B) = 1.022, *p* < 0.001), a positive status for HCV antibodies (Exp(B) = 1.866, *p* < 0.001), receiving an organ from a deceased donor (Exp(B) = 0.576, *p* < 0.001) and increased number of broad antigen mismatches (Exp(B) = 1.161, *p* < 0.001).

The Cox regression analysis for overall survival (Table A2c) found that several factors significantly impact overall survival rates. These factors include: older recipient age (Exp(B) = 1.028, *p* < 0.001), older donor age (Exp(B) = 1.012, *p* < 0.001), a positive Hepatitis C virus antibody status (Exp(B) = 1.700, *p* < 0.001), receiving an organ from a deceased donor (Exp(B) = 0.635, *p* < 0.001), and broad mismatches in major mismatched antigens (Exp(B) = 1.105, *p* < 0.001).

In summary, Cox regression analysis identified significant factors impacting patient survival, graft survival, and overall survival in kidney transplant recipients, including older recipient and donor age, positive Hepatitis C status, deceased donor organs, and broad antigen mismatches, with further validation using an Accelerated Failure Time (AFT) model due to unmet proportional hazards assumptions.

### 3.6. Accelerated Failure Time Model

The study employed AFT regression models to analyse how various independent variables relate to the duration until death (Table 3a), graft failure (Table 3b), and the combined event of graft failure and death (Table 3c).

The recipient’s age and a coinfection with the Hepatitis C virus significantly reduced the patient survival. For every one-year increase in recipient age at the time of transplantation, there was a 4.5% reduction in the patient’s survival time (*p* < 0.001).

The examination of various factors within the context of graft survival indicated that donor age, coinfection with HCV, donor type, and the quantity of broad antigen mismatches significantly influenced graft survival. Living donor type exhibited a highly significant (*p* < 0.001) positive impact on the time to graft failure. Specifically, a graft from a living donor was linked to a 3.8-fold increase in graft survival compared to grafts from deceased donors. All other mentioned variables showed a negative impact on graft survival.

It was observed that older recipient or donor age, coinfection with HCV, and an increased number of broad antigen mismatches all displayed a significant negative impact on both patient and graft survival, resulting in decreased overall survival time. For each additional year in recipient age at the time of transplantation, there was a 2.8% reduction in the overall survival time. Each one-year rise in donor age caused a 1.6% reduction in the overall survival time. Conversely, the presence of a living donor type notably extended the time to the event by a factor of 2.1, contributing to an increased overall survival time.

Overall, AFT regression models revealed that older recipient and donor age, HCV coinfection, and increased number of broad antigen mismatches significantly reduced patient survival, graft survival, and overall survival, while receiving a graft from a living donor positively impacted graft survival and overall survival. Hepatitis B status however showed no significant impact on outcomes in this regression models.

## 4. Discussion

This study provides new insights into the ongoing debate on the impact of different HBV serostatuses on outcomes following kidney transplantation. Our study did not find a significant association between non-active or chronic HBV infection and patient or graft survival after kidney transplantation. Although, Kaplan–Meier curves and RMST analysis initially suggested significant differences in patient and graft survival between patients with chronic or non-active HBV infection and those who tested negative, adjustment for other covariates in the multivariate models attenuated these associations. This suggests that the apparent influence of HBV status on transplant outcomes may be confounded by other factors and highlights the importance of adjusted models in general.

Previous studies have frequently reported inferior outcomes for kidney transplant recipients with chronic HBV infection [20,21,22,23,24]. However, our results indicate that once relevant variables—including recipient and donor age, donor type, CMV antibodies, cold ischemia time, HCV coinfection, and immunological mismatches—are accounted for, HBV status alone does not significantly impact outcomes. This aligns with some prior research that found significant differences in crude survival rates without adjusting for confounding factors in multivariate analyses. For instance, Ahn HJ et al. [22] reported significantly lower survival rates for HBsAg-positive kidney transplant recipients compared to HBsAg-negative recipients, with a 10-year patient survival rate of 64.4% versus 88.2%, respectively. However, no multivariate analysis was performed in the study, leaving open the possibility that the observed differences in survival times could be attributed to other confounding factors like antiviral treatment, immunosuppression, comorbidities or era. Similarly, Lee et al. [29] found that the 10-year survival rate was significantly higher in patients who were HBsAg-negative (82.8%) compared to those who were HBsAg-positive (51.4%) (*p* < 0.005), but their multivariate analysis revealed results comparable to ours, with HBV status showing no or only borderline significance in the modern era of antiviral therapy.

Historically, inferior outcomes in HBV-infected transplant recipients were thought to be driven by an increased risk of liver-related mortality, exacerbated by immunosuppressive therapy, which can enhance viral replication and accelerate liver disease progression [30,31,32,33]. However, significant advances in HBV management, including the widespread use of antiviral therapy, have substantially reduced complications such as hepatocellular carcinoma (HCC) [34,35]. Several studies have demonstrated that antiviral therapy improves post-transplant outcomes in HBV-positive patients [22,36]. However, such therapy was not routinely administered to transplant recipients with a history of HBV infection in many previous studies. More recent studies analyzing the impact of HBV status align with our findings, reporting no significant effect on post-transplant outcomes [37]. This suggests that prognosis has improved with the broader use of antiviral therapy. Therefore, more contemporary outcome studies are needed to address the impact of HBV infection on post-transplantation outcomes today. In a study by Reddy et al., the outcomes of hepatitis B virus (HBV)-positive kidney transplant recipients were evaluated in the context of modern antiviral therapy availability [38]. The authors report that, between 2001 and 2007, HBV-infected renal recipients did not exhibit a higher risk of kidney failure or death compared to HBV-negative recipients. However, these HBV-positive patients remained at a higher risk for liver failure.

To our knowledge, this is one of the first studies to investigate the long-term (>10 years) impact of non-active HBV infection on patient and graft survival after kidney transplantation. Previous research has shown that patients with resolved HBV infection may still harbor low levels of viral DNA, which can persist for years after acute infection [39] and may reactivate under conditions of immunosuppression [40,41]. Nonetheless, studies specifically evaluating the long-term survival implications of this phenomenon are extremely limited [27].

One of the strengths of our study is the large patient cohort and extended study period of over 20 years, allowing for a robust assessment of long-term outcomes. The use of a digital transplant database enabled comprehensive analysis with a wide range of clinical variables, adding depth to our findings. Additionally, data completeness was high, with less than 0.6% missing data for analyzed variables, and loss to follow-up was minimal. Nevertheless, as with all retrospective studies, certain limitations must be acknowledged. Notably, our dataset lacked detailed information on antiviral therapy and HBV vaccination status, both of which could significantly influence patient outcomes. Given the established benefits of antiviral therapy, the absence of these data limits our ability to fully evaluate its impact. Additionally, data on HBV-DNA levels and hepatic complications such as liver failure were unavailable, preventing a more detailed assessment of viral activity and its impact on the liver. Furthermore, apart from Hepatitis C infection and CMV infection, we lacked information on other viral coinfections, such as Hepatitis D or EBV, which may influence post-transplant prognosis. These limitations are due to the long study period and changes in the routine assessment practices over time. Lastly, we did not specifically analyze the impact of race on outcomes, as race was not included in our database, and the majority (>95%) of our patient cohort in Germany is Caucasian.

While our study suggests that chronic and non-active HBV infection may not independently impact graft and patient survival after adjustment for confounders, this does not rule out the potential clinical relevance of HBV status. HBV-positive transplant recipients still require close monitoring due to the risk of viral reactivation, particularly under long-term immunosuppression [41,42]. Previous studies have identified the absence of anti-HBs antibodies and prolonged high-dose steroid use as independent risk factors for HBV reactivation in kidney transplant recipients with resolved infection [43]. Additionally, pre-transplant anti-HBs negativity has been associated with increased reactivation risk, whereas post-transplant HBV vaccination has shown promising results in improving seroconversion rates and should be considered as a preventive strategy [44].

Expanding the scope of research to include multicenter, prospective studies with diverse geographic and demographic populations would help validate our findings in different settings and improve their generalizability. Furthermore, investigating the interplay between HBV status, emerging antiviral therapies, and transplant outcomes could provide further guidance for optimizing care in this patient group.

## 5. Conclusions

Our large single-centre study suggests that, after adjusting for key confounders, chronic and non-active HBV infections are not independently associated with inferior patient or graft survival in kidney transplant recipients within the current treatment landscape. Consequently, we conclude that HBV-positive kidney transplant recipients, regardless of whether the infection is chronic or non-active, do not experience worse outcomes and do not require any special treatment beyond standard immunosuppressive protocols and the regular antiviral therapy that patients with chronic infection are already receiving. This approach could simplify clinical decision-making, reduce unnecessary treatments, and optimize healthcare resource use. However, given the retrospective nature of our study and the lack of detailed data on antiviral treatment, further prospective, multicenter studies are needed to confirm our findings. These studies should focus on refining management strategies for HBV-positive transplant recipients, with an emphasis on adopting individualized treatment plans based on each patient’s specific risk profile, including factors such as comorbidities and age, which may influence post-transplant outcomes.

## Figures and Tables

**Table 1:** Demographic features and perioperative parameters of the recipients and kidney donors grouped by Hepatitis B status. Values are presented as mean and (standard deviation) for continuous variables, absolute numbers and (percentage) for categorical variables.

Characteristic	All Patients	Patients by Hepatitis B Group		
	N = 2490	Hepatitis B Negative, N = 2197	Non-Active Hepatitis B, N = 218	Chronic Hepatitis B, N = 75
**Age**	51 (14)	51 (14)	53 (13)	46 (10)
**Gender**				
Female	961 (39%)	844 (38%)	94 (43%)	23 (31%)
Male	1529 (61%)	1353 (62%)	124 (57%)	52 (69%)
**Height**	1.71 (0.10)	1.72 (0.10)	1.68 (0.11)	1.71 (0.10)
**Blood group**				
0	816 (33%)	710 (32%)	72 (33%)	34 (45%)
A	1116 (45%)	995 (45%)	93 (43%)	28 (37%)
AB	171 (6.9%)	158 (7.2%)	11 (5.0%)	2 (2.7%)
B	387 (16%)	334 (15%)	42 (19%)	11 (15%)
**Hepatitis B status**				
Hepatitis B negative	2197 (88%)			
Non-active Hepatitis B	218 (8.8%)			
Chronic Hepatitis B	75 (3.0%)			
**Anti-HBs level (mlU/mL)**				
<100	962 (57%)	856 (57%)	70 (46%)	36 (95%)
≥100	737 (43%)	652 (43%)	83 (54%)	2 (5.3%)
**Cytomegalovirus antibodies**				
Negative	931 (38%)	872 (40%)	41 (19%)	18 (24%)
Positive	1543 (62%)	1310 (60%)	176 (81%)	57 (76%)
**Hepatitis C virus antibodies**				
Negative	2352 (95%)	2126 (97%)	172 (79%)	54 (77%)
Positive	132 (5.3%)	70 (3.2%)	46 (21%)	16 (23%)
**Underlying disease**				
Acute kidney failure	94 (4.0%)	88 (4.2%)	4 (1.9%)	2 (2.7%)
Chronic kidney disease	227 (9.6%)	202 (9.7%)	19 (9.2%)	6 (8.2%)
Congenital malformation	464 (20%)	419 (20%)	35 (17%)	10 (14%)
Diabetic nephropathy	159 (6.7%)	136 (6.5%)	22 (11%)	1 (1.4%)
Glomerulonephritis	564 (24%)	492 (24%)	51 (25%)	21 (29%)
Hypertensive nephropathy	197 (8.4%)	172 (8.3%)	21 (10%)	4 (5.5%)
IgA nephropathy	214 (9.1%)	188 (9.0%)	18 (8.7%)	8 (11%)
Other	302 (13%)	272 (13%)	21 (10%)	9 (12%)
Pyelonephritis	138 (5.8%)	110 (5.3%)	16 (7.7%)	12 (16%)
**Type of dialysis**				
Haemodialysis	2102 (85%)	1841 (84%)	195 (90%)	66 (89%)
Peritoneal dialysis	200 (8.1%)	184 (8.4%)	12 (5.6%)	4 (5.4%)
Preemptive transplant	175 (7.1%)	162 (7.4%)	9 (4.2%)	4 (5.4%)
**Time on dialysis**	6.5 (5.6)	6.2 (5.1)	9.0 (8.2)	10.6 (8.4)
**Residual diuresis**	731 (821)	749 (826)	602 (778)	530 (736)
**Era of transplantation**				
1990–1999	166 (6.7%)	130 (5.9%)	12 (5.5%)	24 (32%)
2000–2009	1342 (54%)	1173 (53%)	129 (59%)	40 (53%)
2010–2019	982 (39%)	894 (41%)	77 (35%)	11 (15%)
**Transplantation number**				
1	2168 (87%)	1961 (89%)	160 (74%)	47 (63%)
2	259 (10%)	197 (9.0%)	40 (18%)	22 (29%)
3	46 (1.9%)	28 (1.3%)	14 (6.5%)	4 (5.3%)
4	11 (0.4%)	6 (0.3%)	3 (1.4%)	2 (2.7%)
**Panel reactive antibodies**				
<5%	1780 (93%)	1577 (94%)	157 (90%)	46 (77%)
5–85%	115 (6.0%)	86 (5.1%)	16 (9.2%)	13 (22%)
>85%	17 (0.9%)	15 (0.9%)	1 (0.6%)	1 (1.7%)
**Donor type**				
Dead donor	1885 (76%)	1647 (75%)	176 (81%)	62 (83%)
Living donor	605 (24%)	550 (25%)	42 (19%)	13 (17%)
**Number of broad antigen mismatches**				
0	392 (16%)	357 (16%)	26 (12%)	9 (12%)
1	169 (6.8%)	157 (7.2%)	5 (2.3%)	7 (9.3%)
2	505 (20%)	456 (21%)	39 (18%)	10 (13%)
3	644 (26%)	574 (26%)	54 (25%)	16 (21%)
4	386 (16%)	320 (15%)	48 (22%)	18 (24%)
5	273 (11%)	230 (11%)	30 (14%)	13 (17%)
6	110 (4.4%)	95 (4.3%)	13 (6.0%)	2 (2.7%)
**Cold ischemia time**	9.3 (6.2)	9.1 (6.1)	10.1 (6.3)	13.6 (7.7)
**Primary function**				
Yes	1617 (67%)	1441 (67%)	133 (61%)	43 (65%)
No	813 (33%)	706 (33%)	84 (39%)	23 (35%)
**Recipient side**				
Left	1078 (44%)	953 (44%)	89 (42%)	36 (50%)
Right	1383 (56%)	1222 (56%)	125 (58%)	36 (50%)
**Follow-up time**	8.1 (5.6)	8.1 (5.5)	7.8 (5.2)	11.0 (7.0)
**Donor age**	52 (15)	53 (15)	51 (16)	46 (15)
**Donor gender**				
Female	1224 (49%)	1074 (49%)	113 (52%)	37 (49%)
Male	1266 (51%)	1123 (51%)	105 (48%)	38 (51%)
**Donor height**	1.72 (0.11)	1.72 (0.11)	1.72 (0.09)	1.70 (0.10)
**Donor weight**	77 (15)	77 (15)	76 (13)	76 (21)
**Donor BMI**	26.2 (11.7)	26.2 (12.3)	25.5 (4.0)	25.8 (5.5)
**Donor blood group**				
0	969 (39%)	844 (38%)	88 (40%)	37 (49%)
A	1046 (42%)	932 (42%)	87 (40%)	27 (36%)
AB	139 (5.6%)	130 (5.9%)	7 (3.2%)	2 (2.7%)
B	334 (13%)	289 (13%)	36 (17%)	9 (12%)

**Table 2:** Outcome measures for all patients and each Hepatitis B group.

Characteristic	All Patients	Patients by Hepatitis B Group		
	N = 2490	Hepatitis B Negative, N = 2197	Non-Active Hepatitis B, N = 218	Chronic Hepatitis B, N = 75
**Death**				
no	1711 (69%)	1541 (70%)	132 (61%)	38 (51%)
yes	779 (31%)	656 (30%)	86 (39%)	37 (49%)
**Transplant failure**				
no	1964 (79%)	1762 (80%)	155 (71%)	47 (63%)
yes	526 (21%)	435 (20%)	63 (29%)	28 (37%)
**Death and/or transplant failure**				
no	1439 (58%)	1312 (60%)	103 (47%)	24 (32%)
yes	1051 (42%)	885 (40%)	115 (53%)	51 (68%)

**Table 3:** (**a**) Accelerated Failure Time Model Estimates for confounding predictors of long-term patient survival; (**b**) Accelerated Failure Time Model Estimates for confounding predictors of long-term graft survival, (**c**) Accelerated Failure Time Model Estimates for confounding predictors of long-term overall survival.

**(a)**		
**AFT Model for Patient Survival**		
**Variables, Exp(B) and** *p***-Values**		
**Variable**	**Exp(B)**	*p* **-Value**
Hepatitis B status-negative vs. non-active Hepatitis B	0.859	0.142
Hepatitis B status-negative vs. chronic Hepatitis B	0.740	0.066
Recipient age-younger vs. older	0.955	0.000
Donor age-younger vs. older	0.997	0.249
Cold ischemia time-shorter vs. longer	0.995	0.407
Hepatitis C virus antibodies-negative vs. positive	0.658	0.001
Donor type-deceased vs. living donor	1.520	0.000
Cytomegalovirus antibodies-negative vs. positive	1.061	0.362
Broad antigen mismatches-one vs. more	0.945	0.007
**(b)**		
**AFT Model for Graft Survival**		
**Variables, Exp(B) and** *p***-Values**		
**Variable**	**Exp(B)**	*p* **-Value**
Hepatitis B status-negative vs. non-active Hepatitis B	0.641	0.176
Hepatitis B status-negative vs. chronic Hepatitis B	0.437	0.097
Recipient age-younger vs. older	1.022	0.013
Donor age-younger vs. older	0.959	0.000
Cold ischemia time-shorter vs. longer	0.978	0.246
Hepatitis C virus antibodies-negative vs. positive	0.278	0.001
Donor type-deceased vs. living donor	3.824	0.000
Cytomegalovirus antibodies-negative vs. positive	1.037	0.858
Broad antigen mismatches-one vs. more	0.749	0.000
**(c)**		
**AFT Model for Overall Survival**		
**Variables, Exp(B) and** *p***-Values**		
**Variable**	**Exp(B)**	*p* **-Value**
Hepatitis B status-negative vs. non-active Hepatitis B	0.804	0.207
Hepatitis B status-negative vs. chronic Hepatitis B	0.615	0.075
Recipient age-younger vs. older	0.972	0.000
Donor age-younger vs. older	0.984	0.000
Cold ischemia time-shorter vs. longer	0.983	0.091
Hepatitis C virus antibodies-negative vs. positive	0.442	0.000
Donor type-deceased vs. living donor	2.124	0.000
Cytomegalovirus antibodies-negative vs. positive	1.027	0.803
Broad antigen mismatches-one vs. more	0.877	0.000

## Data Availability

Data available on request due to restrictions, e.g., privacy or ethical.

## Abbreviations

The following abbreviations are used in this manuscript:

HBV	Hepatitis B virus
HBsAg	Hepatitis B surface antigen
HbcAg	Hepatitis B core antigen
Anti-Hbc	Hepatitis B core antibody
TBase	Transplant database
KM	Kaplan–Meier
AFT	Accelerated Failure Time
RMST	Restricted Mean Survival Time
HCV	Hepatitis C virus
CMV	Cytomegalovirus
CI	Confidence Interval

## Appendix A

### Kaplan–Meier-Analysis

**Table A1:** (**a**) Cumulative 5-, 10-, and 20-year survival rates of kidney transplant recipients by Hepatitis B status. (**b**) Cumulative 5-, 10-, and 20-year graft survival rates of kidney transplant recipients by Hepatitis B status. (**c**) Cumulative 5-, 10-, and 20-year overall survival rates of kidney transplant recipients by Hepatitis B status.

**(** **a)**			
**Hepatitis B Status**	**5-Year Survival**	**10-Year Survival**	**20-Year Survival**
Hepatitis B negative	87.2%	70.9%	42.5%
Non-active Hepatitis B	81.8%	58.8%	31.3%
Chronic Hepatitis B	82.2%	62.1%	37.1%
**(b)**			
**Hepatitis B Status**	**5-Year Graft Survival**	**10-Year Graft Survival**	**20-Year Graft Survival**
Hepatitis B negative	87.1%	76.5%	60.1%
Non-active Hepatitis B	84.5%	64.0%	52.3%
Chronic Hepatitis B	84.1%	75.7%	32.6%
**(c)**			
**Hepatitis B Status**	**5-Year Overall Survival**	**10-Year Overall Survival**	**20-Year Overall Survival**
Hepatitis B negative	78.6%	59.0%	30.0%
Non-active Hepatitis B	72.8%	44.0%	26.1%
Chronic Hepatitis B	68.7%	50.9%	15.3%

## Appendix B

### Cox Regression Analysis

**Table A2:** (**a**) Multivariate Cox Analysis of factors associated with patient death; (**b**) Multivariate Cox Analysis of factors associated with graft failure; (**c**) Multivariate Cox Analysis of factors associated with graft failure and patient death.

**(a)**				
**Cox Regression for Patient Survival**				
**Variables, Exp(B) and** *p***-Values**				
**Variable**	**Exp(B)**	**CI Low**	**CI High**	*p* **-Value**
Hepatitis B status-negative vs. non-active	1.257	0.990	1.594	0.060
Hepatitis B status-negative vs. chronic	1.379	0.947	2.009	0.094
Recipient age-younger vs. older	1.066	1.057	1.074	0.000
Cold ischemia time-shorter vs. longer	0.999	0.985	1.013	0.899
Hepatitis C virus antibodies-negative vs. positive	1.542	1.146	2.074	0.004
Donor type-deceased vs. living donor	0.554	0.416	0.738	0.000
Cytomegalovirus antibodies-negative vs. positive	0.945	0.811	1.100	0.466
Broad antigen mismatches-one vs. more	1.079	1.027	1.133	0.003
Donor age-younger vs. older	1.004	0.998	1.009	0.202
**(b)**				
**Cox Regression for Graft Survival**				
**Variables, Exp(B) and** *p***-Values**				
**Variable**	**Exp(B)**	**CI Low**	**CI High**	*p* **-Value**
Hepatitis B status-negative vs. non-active	1.263	0.990	1.594	0.108
Hepatitis B status-negative vs. chronic	1.413	0.947	2.009	0.096
Recipient age-younger vs. older	0.994	1.057	1.074	0.127
Cold ischemia time-shorter vs. longer	1.009	0.985	1.013	0.269
Hepatitis C virus antibodies-negative vs. positive	1.866	1.146	2.074	0.000
Donor type-deceased vs. living donor	0.576	0.416	0.738	0.000
Cytomegalovirus antibodies-negative vs. positive	0.994	0.811	1.100	0.948
Broad antigen mismatches-one vs. more	1.161	1.027	1.133	0.000
Donor age-younger vs. older	1.022	0.998	1.009	0.000
**(c)**				
**Cox Regression for Overall Survival**				
**Variables, Exp(B) and** *p***-Values**				
**Variable**	**Exp(B)**	**CI Low**	**CI High**	*p* **-Value**
Hepatitis B status-negative vs. non-active	1.200	0.990	1.594	0.084
Hepatitis B status-negative vs. chronic	1.360	0.947	2.009	0.051
Recipient age-younger vs. older	1.028	1.057	1.074	0.000
Cold ischemia time-shorter vs. longer	1.008	0.985	1.013	0.181
Hepatitis C virus antibodies-negative vs. positive	1.700	1.146	2.074	0.000
Donor type-deceased vs. living donor	0.635	0.416	0.738	0.000
Cytomegalovirus antibodies-negative vs. positive	0.991	0.811	1.100	0.894
Broad antigen mismatches-one vs. more	1.105	1.027	1.133	0.000
Donor age-younger vs. older	1.012	0.998	1.009	0.000
